# Supplementary material for: Structural Analysis of the Menangle Virus P Protein Reveals a Soft Boundary between Ordered and Disordered Regions
Source: Viruses. 2021 Aug 31;13(9):1737. doi: 10.3390/v13091737 (PMC8472933; doi:10.3390/v13091737)
Supplement: Supplementary file 1 [file viruses-13-01737-s001.zip › viruses-1337589-supplementary.pdf]

Figure S1. Large zone SEC analysis of CC-L-BD. (A) The leading edge of a typical large zone elution profile for CC-L-BD (protein concentration in the plateau region = 45 μM). The midpoint of the leading edge was determined by numerical integration and used to compute a mass average partition coefficient for the protein (B) Mass average partition coefficients for protein CC-L-BD (hollow circles) as a function of plateau protein concentration. The solid line shows the predictions of a dimer-tetramer model (Eqns. 2-4, main text) with parameters σ_{P4}=0.22, σ_{P2}=0.51 and equilibrium dissociation constant $K_D = 1/K_A =$ 0.058 μM.

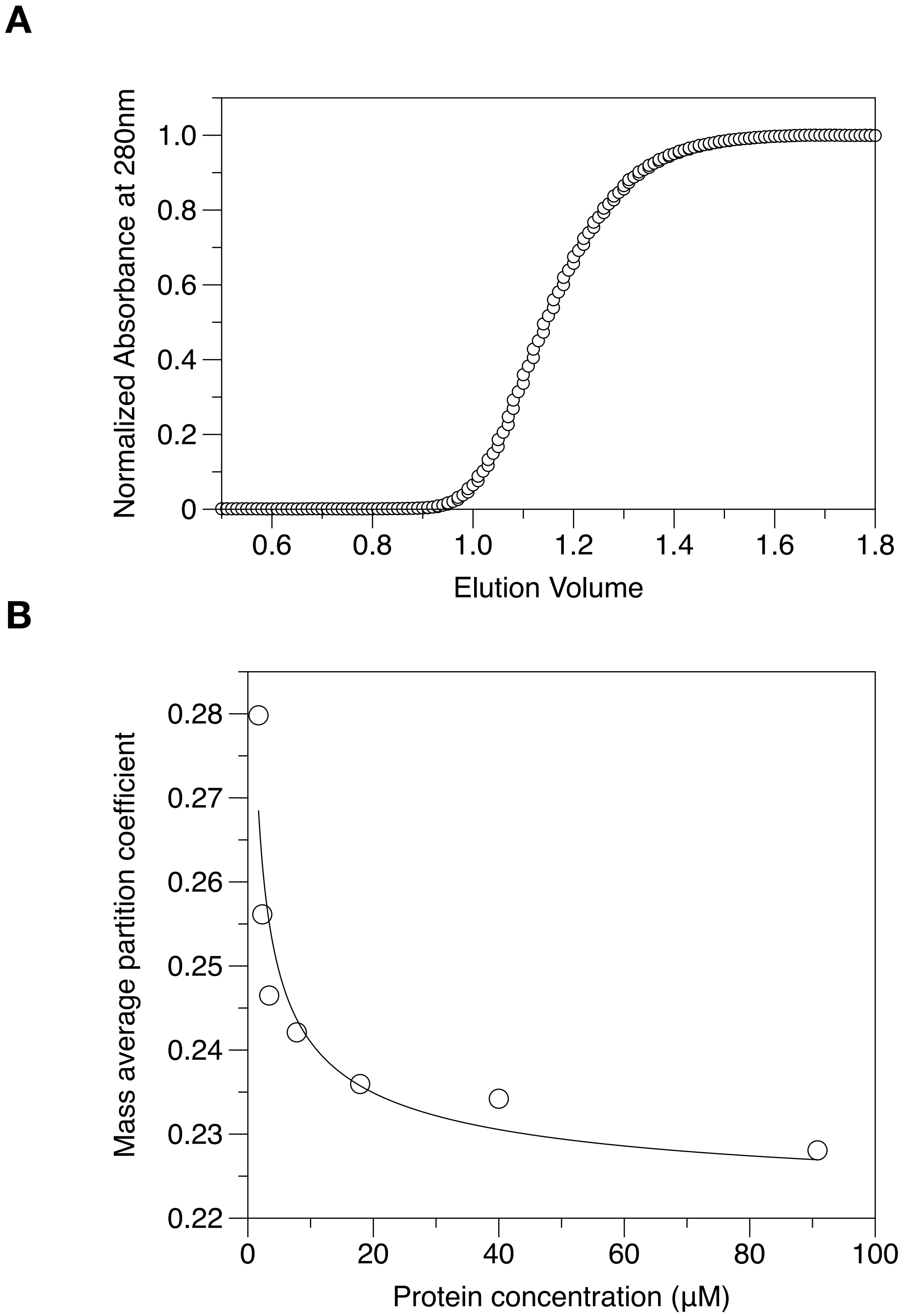

Figure S2. Relative estimates for the forward scattering intensity I(0) for proteins CC-L-BD, L-BD and BD, as a function of protein concentration. The estimates (hollow circles) were derived from Guinier analysis and are shown together with the least squares linear fit (solid lines).

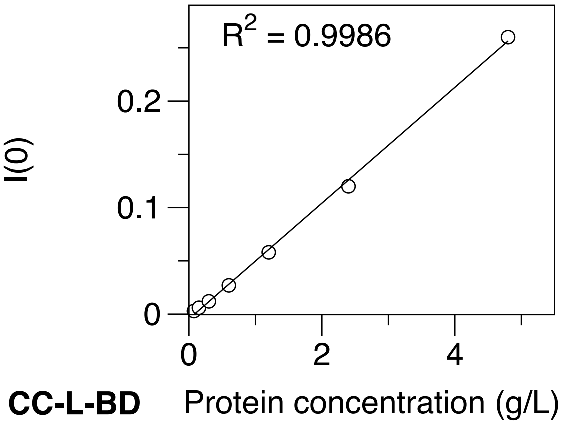

CC-L-BD

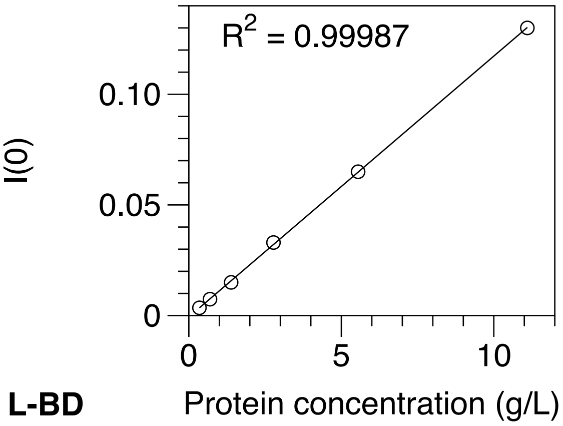

L-BD

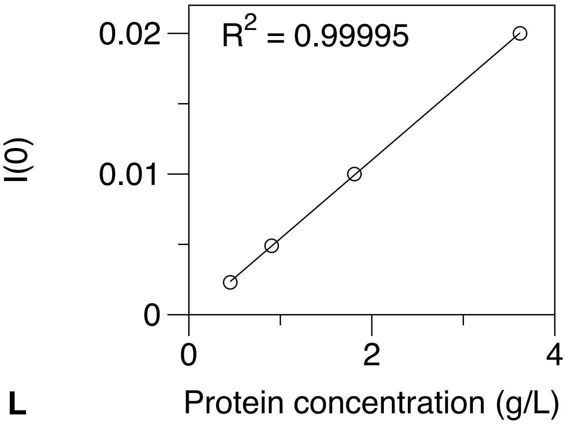

L

Figure S3. Multiple sequence alignment of the P protein from the *Ortho* and *Pararubulaviruses.* The alignment was performed using the Clustal Omega algorithm and results are displayed using BoxShade. Where there is more than 50% identity at a position in the alignment, black shading indicates the consensus residue, while grey shading indicates similarity to the consensus residue.

The abbreviations and the NCBI accession codes are as follows. **SV-41**: Simian virus 41 (NC_006428); **HPIV-2**: Human parainfluenza virus 2 (NC_003443); **PIV-5**: Parainfluenza virus 5 (NC_006430); **MuV**: Mumps virus (NP_054708); **PorPV:** Porcine rubulavirus (NC_009640); **MapV**: Mapuera virus (NC_009489); **HPIV-4b**: Human parainfluenza virus 4b (AB543337); **HPIV-4a**: Human parainfluenza virus 4a (NC_021928); **ThkPV-3**: Tuhoko virus 3 (NC_025350); **SosPV**: Sosuga virus (NC_025343); **ThkPV-2**: Tuhoko virus 2 (NC_025348); **AchPV-1**: Achimota virus 1 (NC_025403); **ThkPV-1**: Tuhoko virus 1 (NC_025410); **AchPV-2**: Achimota virus 2 (NC_025404); **TioPV**: Tioman virus (NC_004074); **TevPV**: Teviot virus (NC_028233); **MenV**: Menangle virus (AF326114).

```
SV-41     1 -MAEEPTYTAEQVNDVVHAGLGTVDFFLSRP-VDGQSSL--GKGSVPP--  44
HPIV-2    1 -MAEEPTYTTEQVDELIHAGLGTVDFFLSRP-IDAQSSL--GKGSIPP--  44
PIV-5     1 MDPTDLSFSPDEINKLIETGLNTVEYFTSQQ-VTGTSSL--GKNTIPP--  45
MuV       1 ---MDQFIKQDETGDLIETGMNVANHFLSAP-IQGTNSL--SKATIIP--  42
PorPV     1 MASSSLSFSDGEITELLETGLGTIESIERMV-AAKGGPDGGIDPESQP--  47
MapV      1 ---MDLTFSPSEIDDLFGTGLDTIQFITDQK-SKQNDAHGSAKD-SPP--  43
HPIV-4b   1 ---MSFEISVEEIDELIETGNLNIDYALKELGATSQPPP--NRPLSQISK  45
HPIV-4a   1 ---MSFEISVEEIEELIETGNLNIDHALKELGATSQSSL--NKPPSQSSR  45
ThkPV-3   1 ---MEPTPSDAEISAWIEKGLATAKHFAPNP-VSSQSSL--GKSTIKK--  42
SosPV     1 ---MDQPPSDAEISAWIERGLATARHFAPGP-VTSQSSL--GKSTIKK--  42
ThkPV-2   1 ---MDPSPSDEEISAWIDKGMDTVQHFISQP-VNPQSSL--GKNTIKS--  42
AchPV-1   1 ---MDTNPSDEEISAWIDKGLDTIQHFVSGP-VTSQSSL--GKSTIKP--  42
ThkPV-1   1 ---MEISPSDEEINIWMDKALDTVDHFTSIP-VNPQSSL--GKNTIKA--  42
AchPV-2   1 ---MDTSPSDAEIAAWIDKGLDTVEHFITSG-SGTPPQH--PRSQREP--  42
TioPV     1 ---MDPSPSDAEISAWIDKGLDTVEHFLSAS-TQSVRSL--GKSTIKP--  42
TevPV     1 ---MDASPSDAEISAWIDKGLDTVEHFLSVA-TPPVRSL--GKSSIKP--  42
MenV      1 ---MDNPPSDAEISAWIDKGLDTVEHFLSVA-TDPARSL--GKSTIKP--  42

SV-41    45 --GITAVLTNAAELKAKTAAA------------------------APVKP  68
HPIV-2   45 --GVTAVLTSAAETKSKPVAA------------------------GPVKP  68
PIV-5    46 --GVTGLLTNAAEAKIQESTN----HQKGS----------VGGGAKPKKP  79
MuV      43 --GVAPVLIGNPEQKNIQYPTT---SHQGS----------KSKGRGS-GA  76
PorPV    48 --GQRGLPTPTPRTTSTPTAAGSASATLEL----------SPEGGAIKKA  85
MapV     44 --QTQNGPDSGPS---DPTQV------QGA----------KPKSHGIYPP  72
HPIV-4b  46 TEENNDETRTSKNSASAEAPAHA-----------------------S-SP  71
HPIV-4a  46 TEGNDGGTKISRNPAPVEAPAHT-----------------------S-TA  71
ThkPV-3  43 --GNTKVLVSSAEQIASSQPAASHTV-KVQAQVHPQQPTASQPCTGARPK  89
SosPV    43 --GNTKQLVDSAEFTAATLAPAGGLQGSMPCSI---PAGQSKAGQGARPK  87
ThkPV-2  43 --GNTKILIKSAEKKSKATKDNV--T-QESAPTPPPRDYQSEKKEVVRPK  87
AchPV-1  43 --GNTRGLVKSAESKSMLAKLAP--P-SSMQPAPPPREDQASSSGGARPK  87
ThkPV-1  43 --GNTKSIIKSAERKARAVPDHANAAPNATVEPATDRPYQTVQ-PVTKPK  89
AchPV-2  43 --QS-QGLQPSKPPRKQPEKQQPATT----PTRDPSLKKAPSKDPGARPK  85
TioPV    43 --GNTEELVAAAEKVAANTAKGILSG---V------------RGTNPDPA  75
TevPV    43 --GNTGELISAAEKLVSNMEGITASS---P------------TMKGPDPA  75
MenV     43 --GKTQELIRSAEKLAGAVVQGGEKG---D----RDNAKKEVTTAAPEPA  83

SV-41    69 KRKKIQHMTPAYTIADNGDPNRLPANTPIANPLIPIERPPGRMTDLDL-- 116
HPIV-2   69 RRKKVISNTTPYTIADNIPPEKLPINTPIPNPLLPLARPHGKMTDIDI-- 116
PIV-5    80 RPKI----------AIVPADDKTVPGKPIPNPLLGLDSTPSTQTVLDL-- 117
MuV      77 RP------------IIVSSSEGGTGGTQVPEPLFAQTGQGGIVTTVYQ-- 112
PorPV    86 PR------------AHPTLPNPLGQEERPGNPLSTFTPVRGSSSTHDP-- 121
MapV     73 IP------------TAPPVPTARHPGSRVDDPVLYDYPRRGKVTTHEP-- 108
HPIV-4b  72 LRSH---------------NEESEPGKQSSDGFSMISNRPQTGMLLMGSD 106
HPIV-4a  72 QRSH---------------NEENESGRQNLDSLSMISNKPQTGTLLMGSD 106
ThkPV-3  90 TKRTATPA-----------PTPSVQQAVKIEPV-YEDIVSNPTHQ--A-- 123
SosPV    88 VKKAAQTR-----------ATPTKPDAPTIEPV-YEDIVSTPGRD--K-- 121
ThkPV-2  88 IRKTQGER-----------PRPLPPIPQQEESI-YEEVSREVVEEDQP-- 123
AchPV-1  88 SKKSVSFQ-----------KPQASQSVISDDAI-YEEVIRPDNDEFQP-- 123
ThkPV-1  90 QPRKARALPCP-----SSAPPVPPRVSEPQEEP-YEELGAPAQEDTPL-- 131
AchPV-2  86 QLSTVRFG-----------PGVTVAPCGPEDSI-YEEVIPGGSTCAGQ-- 121
TioPV    76 TRPKEKQK-----------GSPVKMQHQEQESV-YEEVIPTESAPLIP-- 111
TevPV    76 VRPKERTK-----------PPQDNAQNQEQNDI-YEEVIPSESTALIP-- 111
MenV     84 VRGKVR-----------------PIDVEPSDNT-YEEVIPSENSKLIP-- 113
```

Figure S3 (continued)

```
SV-41     117 ATGTVTQGTYKGVELAKAGKNALLTRFSSGPSL--TDQASSKDPNFKRGG 164
HPIV-2    117 VTGNITEGSYKGVELAKLGKQTLLTRFTSNEPV--SSAGSAQDPNFKRGG 164
PIV-5     118 SGKTLPSGSYKGVKLAKFGKENLMTRFIEEPRE--NPIATSSPIDFKRGA 165
MuV       113 DPTIQPTGSYRSVELAKIGKERMINRFVEKPRT--ST----PVTEFKRGG 156
PorPV     122 LPGSRPGSEVYEGDLMARARSELVTRWSDEEGD--PVPTRVLQSTFKRGG 169
MapV      109 KESSQADGYEYDSYLAQNAKTNILKRWTDVSGD--VEPIPMNPEVFKRGG 156
HPIV-4b   107 TQSPSPSKTYQGLI--LDAKKRALNEPRRNQKTTNEHGNTNDTWIFKRGG 154
HPIV-4a   107 TQLPSPSKTYQGLI--LDAKKRALNEPRRDQKITNEHGSMNDTRIFKRGG 154
ThkPV-3   124 ENAPLI--------ATQSSAKQSLLCTEPLPSQP--SSATRCGEQSFKRGG 164
SosPV     122 AQVQLP--------SPKLSAKDKLLSAPPLQQHS--NIPTGPEGQNFKRGG 162
ThkPV-2   124 LLQQQQ--------AHVLKGKQKILSTSPVNQEP--DLPTGPGGQGFKRGG 164
AchPV-1   124 LLQKSP--------SPEQKAKDRLLNTVIMGDHQ--PPSTSHSGQPFKRGG 164
ThkPV-1   132 LKQAVP--------QSPSSGKLKSSQLPPLDNSNDSSNPSLSDQNIFKRGA 174
AchPV-2   122 KQPGKD--------DRKTKAKNRILSMQVDDTVV--GGVDQGMPSHFKRGG 162
TioPV     112 KTT--P--------KKPPRNKEKVMSMMALSPPD--ESLDETHEAVFKRGG 150
TevPV     112 KSA--P--------KKPSRNKEKVMSMMALSPPE--DNSAKEKPSVFKRGG 150
MenV      114 PVT--P--------KKPPRHKDRIMSMMPLQSDK--QLTESMESQVFKRGG 152

SV-41     165 EKLT---------------DATKADIGGSGASPGSETKLRFMSGAIQHVPQ 200
HPIV-2    165 ELIE---------------KEQEATIGENGVLHGSEIRSKSSSGVIPGVPQ 200
PIV-5     166 GIPA--------------------GSIEGSTQSDGWEMKSRSLSGAIHPVLQ 197
MuV       157 PGAAAQ-----------GQTIQEEGIDGNGASAGSKERSGSLSGATPYAHL 196
PorPV     170 PTGKEL-------------IPANQATVENIASGGSVGPSGSSNGATQHVPQ 207
MapV      157 QTSPSP-------------QIPIQVIAENGALGGLGQQSRSWSGATPPALQ 194
HPIV-4b   155 NIATKKEAWVTQNQRSKIQSSFQDIEESTRFHGSMEEPQYQSGAIHVAHQ 204
HPIV-4a   155 NIDTRKEAWVTQNQRSRTQPPLQDIEESTRFHGLTEEPQYPSGAIHAAHQ 204
ThkPV-3   165 DFLPVP---------PGRDPTVTQDTDENLILFGAQENLRSENGATQPAPQ 206
SosPV     163 CPQEAP---------PLLEETPIQDTDESSILYGEEDHSQCESGAIQPAVQ 204
ThkPV-2   165 LGLISQ---------QQDSSTYDQGIDESLTSDGMATNLVLESGATLAVPL 206
AchPV-1   165 FGVTGV---------QQQVQNSRDAIGESLILDGLEEDPMSVNGATPYVPQ 206
ThkPV-1   175 YPRVPD-----------GMNKKLHVTGESSTLSGVGAVQSSLSGATQSAPP 214
AchPV-2   163 DTASMS-----------ALKGSGIAIAENTPSIGAQAALESSSGATQAVSQ 202
TioPV     151 VPPPHG-----------RGATGQGDTGGKSPSAGQPEPRGSQNGATQYVTQ 190
TevPV     151 IPPPHG-----------PAATDKGATGGRLQSAGQQGSHESQNGATQYVTQ 190
MenV      153 KDLRHG-----------PSDIGPGAIGGRSQLTGLAGGRESQSGATQYVTQ 192

SV-41     201 LLPLTASSPVLVEPAPIGAENVKEIIEILRGLDLRMQSLEGKVDKILATS 250
HPIV-2    201 SRPQLASSPAHADPAPASAENVKEIIELLKGLDLRLQTVEGKVDKILATS 250
PIV-5     198 SPLQQGDLNALVTSVQSLALNVNEILNTVRNLDSRMNQLETKVDRILSSQ 247
MuV       197 SLPQQDSTPANVGIAPQSAISANEIMDLLRGMDARLQHLEQKVDKVLAQG 246
PorPV     208 YPWNQDTTNAPVAPAPQSALNVPEIMELLKAIEGRMMALEMKVDRVLAQG 257
MapV      195 LQQHPDTMNVSVESALRSAPDVSEIMDMLRRLDARIGSVEAKMDRILAVG 244
HPIV-4b   205 SNQLPPSKNVHVEDVPKFANYALEILDAIKALEVRLDRIEGKVDKIMLTQ 254
HPIV-4a   205 SNQLPLSKNAHVEDVPKFANYASEILDAIKALEVRLDRIEGKVDKIMLTQ 254
ThkPV-3   207 SLQSPEDISAVVGNALESANSVREIIRYLKVMEAKMTQIEWKVDKVLAQN 256
SosPV     205 SHQSPGDITADVVSALESANSVKEIIRYLKVMENKMNQLEWKIDKVLAQN 254
ThkPV-2   207 SDQLQCDIPALVENALQSAPCVKEILRYLRVLENRFNQMESKIDKIISHQ 256
AchPV-1   207 SLQSHGDLHAAVETALMSAPCVREILRYLKLLENRFNQMDWKLDKLIGQQ 256
ThkPV-1   215 SLPIQDDNPVGAGDAQVFANSVKEITTILQGMEARMSQIEWKVDKLLAQQ 264
AchPV-2   203 LQIQEQESPVDVATVPELAPCVKEILRYLKVIETRMTQLEWKVDKIIGQN 252
TioPV     191 YPNSPTGEPAGAGAVQMSAPCVREIMHYLQTLETRIANLDWKVDKLLSQQ 240
TevPV     191 YLNPRTEDPVGADSAQMSALCVREIMHYLQTLETRITNLDWKVDKLLSQQ 240
MenV      193 SPSQPSEVAADVETAPASAPYVKEIIHYLQTLETRINNLDWKVDKILSQQ 242
```

Figure S3 (continued)

```
SV-41    251 ATITALKNEVTSLKANVATVEGMMTTMKIMDPSTPTNVPVEKIRKNLKDT 300
HPIV-2   251 ATIINLKNEMTSLKASVATMEGMITTIKIMDPSTPTNVPVEEIRKSLHNV 300
PIV-5    248 SLIQTIKNDIVGLKAGMATLEGMITTVKIMDPGVPSNVTVEDVRKTLSNH 297
MuV      247 SMVTQIKNELSTVKTTLATIEGMMATVKIMDPGNPTGVPVDELRRSFSDH 296
PorPV    258 SVLTQIKNEVTTLKATTATIEGLITTVRIMDPGVPSNMTAQIARNQIAEV 307
MapV     245 ATVNQIKNEVSSLKSITATIEGMITTVRIMDPGTPSHMSATEVRRQLSDV 294
HPIV-4b  255 NTIQQTKNDTQQIKGSLATIEGLITTMKIMDPGVPSKVSLRSLNKGPEQV 304
HPIV-4a  255 NTIQQTKNDTQQIKGSLATIEGLITTMKIMDPGVPSKVSLRSLNKESEQV 304
ThkPV-3  257 SLIQQVRNEQLVLKASMATIEGLMTTIKIMDPGVGPGATAAQAKRLFKEA 306
SosPV    255 NIIQQIRNEQMVLKAGMATLEGLITTIKIMDPGVGTGADAAQAKRAFKDV 304
ThkPV-2  257 NILTQIRNEQLGLKASMAMLEGMITSIKIMDPGVGPGATAAQAKKLFKEV 306
AchPV-1  257 STITQIRNEQMGLKANMAMIEGLITSIKIMDPGVGTGANAAQAKKLFKEV 306
ThkPV-1  265 STITQIRNEQVALKAQMATIEGLLATVKIMDPGAVSSTTANQAKKYFTES 314
AchPV-2  253 NMIQQIRNDQTALKANMATIEGILTTIKIMDPGVPTSSNASQVKKLFKES 302
TioPV    241 TTITQIKNDQHTIKASLATIEGLITTIKIMDPGVGPGATASQAKRIFKEA 290
TevPV    241 STITQIKNDQHTIKASLATIEGLITTIKIMDPGVGPGATAAQAKKIFKEV 290
MenV     243 SVITQIKHEQHAIKAGIATLEGLITTIKIMDPGVGDGATAAKSKRLFKEA 292

SV-41    301 PVIISGPLSESHITEGSDMIVLDELARPSLSSTKKIV-RRPEPKKD-LTG 348
HPIV-2   301 PVVIAGPTSGGFTAEQVILISMDELARPTLSSTKRIT-RKPESKKD-LTG 348
PIV-5    298 AVVVPESFNDSFLTQSEDVISLDELARPTATSVKKIV-RKVPPQKD-LTG 345
MuV      297 VTIVSGPGDVSFSSGEEPTLYLDELARPVPKPRPAKQ-PKPQPVKD-LAG 344
PorPV    308 PLIVTGPGPVPQYKRDTDLIVLDELARPSIAPLPAAQTQKPAPAKQATDG 357
MapV     295 PLVISGPGPVPQLDPRRDLIALDELARPKVVQSPPVASPAPSMASQLSDG 344
HPIV-4b  305 PIIVTGTGDVSKFVDQDNTITLDPLARPILSGTKQIT-DERRAGVR-IDA 352
HPIV-4a  305 PIIVTGNGDVSKFVDQDNTITLDSLARPILSGTKQKT-DERRAGVR-IDA 352
ThkPV-3  307 PVVVSGPIVGDNDLIFEDKIEISSLGKPQKVAPQPKK-RLVTSEAD-IAG 354
SosPV    305 PVVVSGPVIGENDLIFESKLEVGNLGRPQKVNPTPRR-RGVPTESE-LAS 352
ThkPV-2  307 PVVISGNSVGATELTEAAELEIQDLGRPVIPQQTPKK-RAVVGDSD-LAS 354
AchPV-1  307 PVVISGPVVGENPLVTATELEIQDLGKPAIPSKGSSK-KAGLTEAD-LAG 354
ThkPV-1  315 CVVVSGPPTGDLELTKAKELFIGDLGTPTPNPPPQPQ-PAQSDSRE-LAG 362
AchPV-2  303 PVVISGPCSADNPLISAGSLQLDELARPSIPTTMAKK-KQVSTDSE-ISG 350
TioPV    291 PVVISGPILGDNPIIGAEAIQLDELARPSPAKPRQVK-QSGPSSSA-IVG 338
TevPV    291 PVVISGPILGENQVIHADTIQLDELARPSPAKGKQAK-TASPNPNA-VIG 338
MenV     293 PVVVSGPVIGDNPIVDADTIQLDELARPSLPKTKSQK-SSAASPAA-LSG 340

SV-41    349 MKLMLIQLANDCMGKPDQKAEIVAKIHAATREAQLLDIKRSIIKSAI- 395
HPIV-2   349 IKLTLMQLANDCISRPDTKTEFVTKIQAATTESQLNEIKRSIIRSAI- 395
PIV-5    346 LKITLEQLAKDCISKPKMREEYLLKINQASSEAQLIDLKKAIIRSAI- 392
MuV      345 RKVMITKMITDCVANPQMKQVFEQRLAKASTEDALNDIKRDIIRSAI- 391
PorPV    358 ARLMVSRMISSCVTNDSARKRFEARLGSCTTMDQIQALKNDVIRYAS- 404
MapV     345 TRIMILQMIKECVSDPLEQSRFETKLSSCTTEDQGKAIKMEILRRAT- 391
HPIV-4b  353 LKITVSEMIRDLFGDCDKSRKLLESINMATTEQDINSIKTNALRSIT- 399
HPIV-4a  353 LKITVSEMIRDLFGDCDKSKKLLESINMATTEQDINLIKTNALRSIT- 399
ThkPV-3  355 YKLTLTKLLKECIPNANQHKKFEDLIASVKNESDFKAAKREIVRAAI- 401
SosPV    353 YKLTLAKLLKDCIPNNTAQSKFLDAIEKIKTEADFKALKREIVRAAV- 399
ThkPV-2  355 YKLTLKNLAKDCIPNAHIQAEFDKKISSIRSENDFKKIKREILRAAT- 401
AchPV-1  355 YKMTLMTLIKDCIPNASMQQEFERRVNQIKNEVDFKALKREILRAAV- 401
ThkPV-1  363 YKMTLNMLAKDCIPNSQQRQPFLKKIETIKSEQDFKKLKREIIRAAV- 409
AchPV-2  351 YRLTLITLISDCIQNPIQQQTFEKKAGLVKTEQEFKKLKREILRAAV- 397
TioPV    339 YKSTLQSLVKECISNPSMRQKFDLAISNIKSEQDFKQVRRDIIRSAT- 385
TevPV    339 YRSTLQSLVKECITNPGLRQKFDVAINSVKTEQDFKQVRRDIIRSAT- 385
MenV     341 YKMTLLALIKECIPNQAKRQKFEMQVGGIRNEQDFKNLRREIIRSAAQ 388
```

Figure S4. Secondary structure propensities for the linker sequence (residues 268-330) (A) Predicted by the algorithm GOR IV from the full length MenV P sequence. (B) Predicted by the algorithm ncSPC (neighbor-corrected Structural Propensity Calculator) based on solution NMR chemical shifts of MenV $P_{267\text{-}388}$ (pH 7, 10 °C). ncSPC scores of 1.0 and – 1.0 would reflect fully formed α- or β-structure, respectively.

A

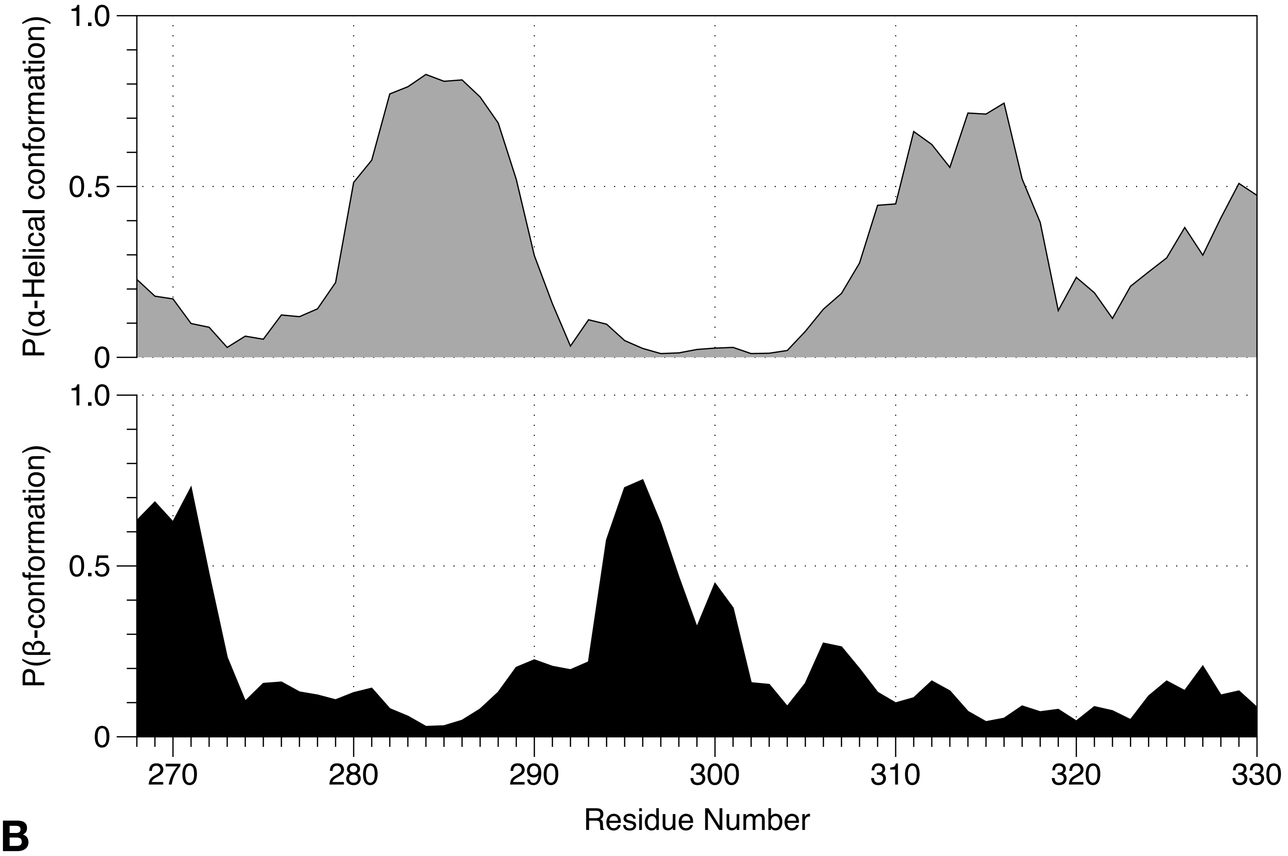

B

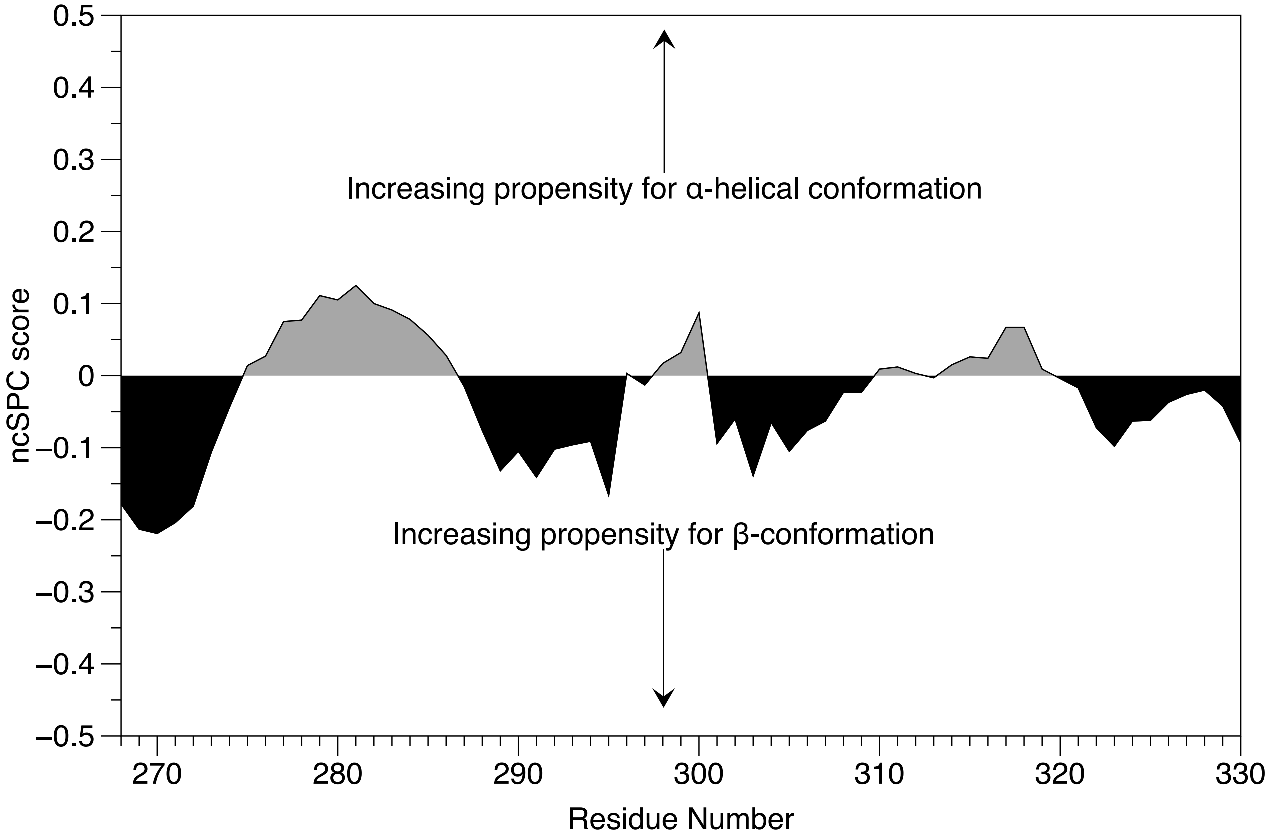

Figure S5. Two ^{1}H-^{1}H sections of a 3D ^{1}H-^{15}N NOESY-HSQC experiment (mixing time 120 ms), showing NOE cross-peaks involving the backbone amide protons of Ser 334 (left) and Ala 337 (right). A well defined NOE is observed between the backbone amide proton of Ser 334 and the side chain methyl protons of Ala 337, consistent with close proximity of S334 N and A337 Cβ in the crystallographic models (interatomic distances 4.3-4.4 Å)

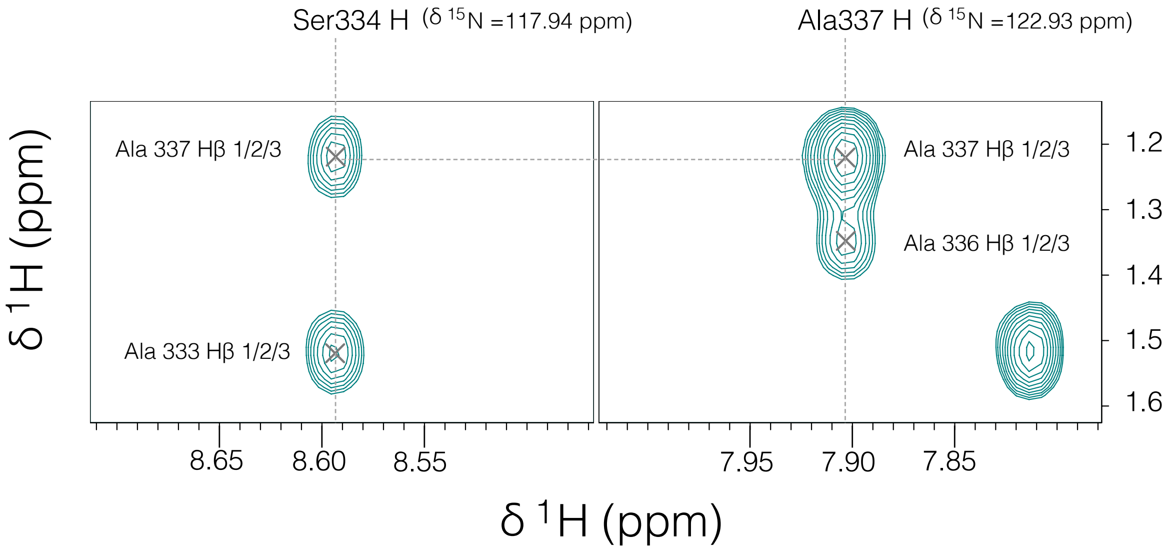

Table S1. Bacterial expression plasmids facilitating production of truncated MenV P proteins for biophysical analysis

Protein	Parental Vector	Cleavable Affinity Tag	Plasmid Name	Detail of insertion into the multiple cloning site of the vector
**MenV $P_{209\text{-}388}$ (CC-L-BD)**	pET15b(+)	polyhistidine	pMW596	*NcoI* **MenV P** *XhoI*<br>CC ATG GGC AGC AGC CAT CAT CAT CAT CAT CAC ACT AGT GAA AAC CTG TAT TTT CAG GCA AGC GCA … GCA GCA CAG TAA CTC GAG<br>Met Gly Ser Ser His His His His His His Thr Ser Glu Asn Leu Tyr Phe Gln **Ala Ser Ala** … **Ala Ala Gln** --- --- –<br>poly-histidine tag TEV recognition seq. **209 210 211 386 387 388**
**MenV $P_{209\text{-}336}$ (CC-L)**	pET15b(+)	polyhistidine	pMW598	As for plasmid pMW596, with a stop codon inserted into the MenV P coding sequence, following codon for Ala 336
**MenV $P_{209\text{-}271}$ (CC)**	pET15b(+)	polyhistidine	pMW597	As for plasmid pMW596, with a stop codon inserted into the MenV P coding sequence, following codon for Ile 271
**MenV $P_{267\text{-}388}$ (L-BD)**	pET41a(+)	GST	pMW591	**GST** **MenV P** *XhoI*<br>… TCG GAT GGC AGT GAC TAC GAT ATC CCA ACT AGT GAA AAC CTG TAT TTT CAG ACC ACC ATC … GCA GCA CAG TAA CTC GAG<br>… **Ser Asp** Gly Ser Asp Tyr Asp Ile Pro Thr Ser Glu Asn Leu Tyr Phe Gln **Thr Thr Ile** … **Ala Ala Gln** --- --- ---<br>spacer sequence TEV recognition seq. **267 268 269 386 387 388**
**MenV $P_{267\text{-}328}$ (L)**	pET41a(+)	GST	pMW652	As for plasmid pMW591, with a stop codon inserted into the MenV P coding sequence, following codon for Gln 328
**MenV $P_{337\text{-}388}$ (BD)**	pET41a(+)	GST	pKY302	**GST** **MenV P** *XhoI*<br>… TCG GAT GGC AGT GAC TAC GAT ATC CCA ACT AGT GAA AAC CTG TAT TTT CAG GCC CTG AGC … GCG GCG CAG TGA CTC GAG<br>… **Ser Asp** Gly Ser Asp Tyr Asp Ile Pro Thr Ser Glu Asn Leu Tyr Phe Gln **Ala Leu Ser** … **Ala Ala Gln** --- --- ---<br>spacer sequence TEV recognition seq. **337 338 339 386 387 388**

The Men V P gene sequence (UniProt ID Q91MK1; Genbank Accession AF326114) was codon-optimized for expression in *E. coli*. To limit chemical reactivity of the protein, a Cys352Ser mutation was introduced into the P coding sequence using site-directed mutagenesis.

Table S2. Bacterial expression plasmids facilitating production of MBP-MenV P fusion proteins for crystallographic analysis.

Protein	Parental Vector	Plasmid Name	Detail of insertion into the multiple cloning site of the vector
**MBP-P$_{321-388}$**	pMALX(A)	pMW604	**MBP** *NotI* **MenV P protein** *HindIII*<br>GCG CAG ACT AAT GCG GCC GCA AGC CTG CCG ... GCA GCA CAG TAA AAG CCT<br>Ala Gln Thr Asn Ala Ala Ala **Ser Leu Pro ... Ala Ala Gln** - - -<br>**321 322 323 386 387 388**
**MBP-P$_{327-388}$**	pMALX(A)	pMW605	**MBP** *NotI* **MenV P protein** *HindIII*<br>GCG CAG ACT AAT GCG GCC GCA AGC CAG AAA ... GCA GCA CAG TAA AAG CCT<br>Ala Gln Thr Asn Ala Ala Ala **Ser Gln Lys ... Ala Ala Gln** - - -<br>**327 328 329 386 387 388**
**MBP-P$_{329-388}$**	pMALX(A)	pMW606	**MBP** *NotI* **MenV P protein** *HindIII*<br>GCG CAG ACT AAT GCG GCC GCA AAA AGC AGC ... GCA GCA CAG TAA AAG CCT<br>Ala Gln Thr Asn Ala Ala Ala **Lys Ser Ser ... Ala Ala Gln** - - -<br>**329 330 331 386 387 388**
**MBP-P$_{330-388}$**	pMALX(A)	pMW607	**MBP** *NotI* **MenV P protein** *HindIII*<br>GCG CAG ACT AAT GCG GCC GCA AGC AGC GCA ... GCA GCA CAG TAA AAG CCT<br>Ala Gln Thr Asn Ala Ala Ala **Ser Ser Ala ... Ala Ala Gln** - - -<br>**330 331 332 386 387 388**

The Men V P gene sequence (UniProt ID Q91MK1; Genbank Accession AF326114) was codon-optimized for expression in *E. coli*. To limit chemical reactivity of the protein a Cys352Ser mutation was introduced into the P coding sequence using site-directed mutagenesis.

Table S3. Media and Buffers used for Protein Purification

Protein	Lysis Buffer	Affinity Media	Affinity Chromatography Wash and Elution Buffers	Ion Exchange media	Ion Exchange chromatography Buffers	Size Exclusion media	Size Exclusion Chromatography Buffer
**$P_{209-388}$ (CC-L-BD)**	12.5 mM MOPS/KOH pH 7.0<br>250 mM NaCl	Talon Metal Affinity Resin [Clontech]	Wash: Lysis Buffer + 50 mM Imidazole<br>Elution: Lysis Buffer + 400 mM imidazole<br><br>Poly-histidine tag cleaved post-elution with TEV protease.	SP Sepharose HP [Cytiva]	12.5 mM MOPS/KOH pH 7.0<br>1mM EDTA<br>50-1000 mM NaCl	Superdex 200 [Cytiva]	12.5 mM MOPS/KOH pH 7.0<br>250 mM NaCl
**$P_{267-388}$ (L-BD)**	12.5 mM MOPS/KOH pH 7.0<br>150 mM NaCl	Glutathione Sepharose 4B [Cytiva]	Wash: Lysis Buffer<br>Elution: Lysis Buffer (following on-column cleavage of GST fusion protein with TEV protease)	SP Sepharose HP [Cytiva]	12.5 mM MOPS/KOH pH 7.0<br>50-1000 mM NaCl	Superdex 75 [Cytiva]	12.5 mM MOPS/KOH pH 7.0<br>150 mM NaCl
**$P_{267-328}$ (L)**	12.5 mM Tris/ HCl pH 8.5<br>150 MM NaCl	Glutathione Sepharose 4B [Cytiva]	Wash: Lysis Buffer<br>Elution: Lysis Buffer (following on-column cleavage of GST fusion protein with TEV protease)	Q Sepharose HP [Cytiva]	12.5 mM Tris/HCl pH 8.5<br>50-1000 mM NaCl	Superdex 75 [Cytiva]	12.5 mM Tris/ HCl pH 8.5<br>150 mM NaCl
**$P_{337-388}$ (BD)**	12.5 mM MOPS/KOH pH 7.0<br>150 mM NaCl	Glutathione Sepharose 4B [Cytiva]	Wash: Lysis Buffer<br>Elution: Lysis Buffer (following on-column cleavage of GST fusion protein with TEV protease)	SP Sepharose HP [Cytiva]	12.5 mM Malic acid/KOH pH 3.7<br>50-1000 mM NaCl	Superdex 75 [Cytiva]	12.5 mM MOPS/KOH pH 7.0<br>150 mM NaCl
**MBP-$P_{329-388}$**	12.5 mM Tris/ HCl pH 8.5<br>150 MM NaCl	Amylose Resin [NEB]	Wash: Lysis Buffer<br>Elution: Lysis Buffer + 40 mM Maltose	Q Sepharose HP [Cytiva]	12.5 mM Tris/ HCl pH 8.5<br>50-1000 mM NaCl	Superdex 75 [Cytiva]	12.5 mM Tris/ HCl pH 8.5<br>150 MM NaCl

Table S4. $^3J_{HN\text{-}H\alpha}$ coupling constants for L-BD (10 °C, pH 7.0) estimated using the ARTSY-J pulse sequence.

residue	$^3J_{HN\text{-}H\alpha}$	error	residue	$^3J_{HN\text{-}H\alpha}$	error	residue	$^3J_{HN\text{-}H\alpha}$	error
**269**	9.57	0.02	**311**	7.23	0.02	**353**	8.77	0.10
**270**	7.24	0.02	**312**	6.74	0.03	**355**	6.03	0.13
**271**	7.63	0.02	**313**	8.24	0.03	**357**	4.45	0.15
**272**	7.17	0.02	**314**	6.52	0.03	**359**	2.72	0.42
**273**	6.44	0.02	**315**	6.36	0.03	**360**	3.05	0.23
**275**	7.30	0.02	**316**	5.54	0.04	**361**	4.00	0.18
**276**	7.45	0.02	**317**	6.95	0.03	**362**	4.86	0.19
**277**	7.49	0.03	**318**	6.08	0.03	**363**	3.68	0.37
**278**	6.94	0.02	**319**	6.72	0.03	**364**	4.43	0.17
**279**	7.45	0.04	**321**	7.11	0.05	**365**	5.13	0.23
**280**	5.80	0.03	**322**	6.78	0.04	**367**	8.19	0.15
**281**	7.00	0.03	**324**	6.70	0.05	**368**	5.97	0.23
**282**	5.50	0.04	**325**	7.23	0.08	**369**	4.28	0.27
**283**	5.28	0.04	**326**	6.30	0.15	**370**	8.86	0.47
**284**	6.58	0.04	**328**	6.94	0.42	**371**	5.94	0.25
**285**	6.48	0.07	**329**	6.26	0.19	**373**	4.71	0.18
**286**	6.90	0.08	**330**	7.04	0.27	**374**	3.34	0.25
**287**	6.32	0.04	**331**	7.29	0.72	**375**	2.54	0.83
**288**	7.18	0.03	**332**	7.34	0.02	**377**	4.07	0.21
**289**	7.17	0.03	**333**	5.76	0.10	**378**	5.14	0.23
**290**	7.04	0.04	**334**	5.05	0.11	**380**	3.86	0.29
**291**	6.25	0.03	**336**	4.36	0.32	**381**	4.76	0.21
**292**	5.56	0.03	**337**	4.83	0.19	**383**	3.67	0.46
**294**	6.20	0.06	**338**	3.52	0.28	**384**	4.23	0.18
**295**	7.28	0.02	**339**	3.30	0.20	**385**	5.20	0.15
**296**	7.69	0.02	**340**	6.89	0.18	**386**	4.71	0.10
**297**	7.11	0.04	**341**	2.94	0.30	**387**	6.42	0.04
**298**	7.63	0.03	**342**	3.13	0.41	**388**	7.63	0.02
**300**	7.47	0.02	**343**	3.39	0.28			
**301**	7.23	0.02	**344**	2.77	0.38			
**302**	6.47	0.04	**345**	4.73	0.37			
**303**	7.09	0.02	**346**	2.90	0.53			
**304**	7.43	0.02	**347**	4.47	0.17			
**306**	7.25	0.03	**348**	3.47	0.31			
**307**	7.83	0.03	**349**	2.33	0.93			
**308**	6.46	0.03	**350**	3.05	0.33			
**309**	5.13	0.03	**351**	5.34	0.15			
**310**	6.87	0.02	**352**	8.32	0.15			

Severe line broadening in the dephased spectrum prevented estimation of $^3J_{HN\text{-}H\alpha}$ for some non-proline residues.
